# Agathin

**Published:** 1894-01-13

**Authors:** 


### NEW DRUGS AND PREPARATIONS.

[We shall be glad to receive, at our Office, 428, Strand, London, W.C., from the manufacturers, specimens of all new preparations and drugs which may be brought out from time to time.]

### AGATHIN.

This substance, one of the most recently introduced[1] of the synthetic remedies, is obtained by the combination of salicylic aldehyde and α-methylphenylhydrazine, and thus it is named salicyl and α-methylphenylhydrazon. It occurs in somewhat greenish white scales, having neither smell nor taste. It is soluble in alcohol and in ether, but not in water.

From its chemical constitution it was believed that this substance would have anodyne properties, and the results of some observers who have employed it in their practice bear out this hypothesis. Thus, Rosenbaum used it first in an intractable case of sciatica in a young man aged 25, who was confined to his bed with the pain in the hip and thighs. After a careful trial of such remedies as hot and cold douching, electricity, massage, &c., without any beneficial results, agathin was given, and in three days' time the patient could walk without much difficulty.

But it is evidently a drug that must be given with caution, for Ilberg[1] has investigated the claims of agathin as an analgesic and anti-rheumatic remedy. He gave it to seventeen different cases—two of facial neuralgia, one in a diabetic patient, and one a case of locomotor ataxia, three cases of sciatica, three of tabes, of which one had laryngeal and gastric crises, four of acute articular rheumatism with endo-and pericarditis, three cases of chronic rheumatism, and two of rheumatic blennorrhagia. He began by giving the remedy three times daily in doses of ·25 of a gramme, and gradually increased it to double this amount repeated five times every twenty-four hours. Usually he gave three doses daily of half a gramme. His results were not altogether encouraging. Of eight cases in the first group of infra-orbital neuralgia, tabes, and sciatica, only one (a case of sciatica) obtained any relief. In this case the pains disappeared after the third day, and on the fourteenth he left the hospital; but as regards the other seven cases the remedy was a complete failure. The same abortive results followed in the cases of acute rheumatism and blennorrhagia. A chronic rheumatic patient was relieved, but it ought to be stated that this improvement was not very marked till after fifteen days' rest in bed.

The disagreeable after-effects are very frequent and extremely variable; more than half of the patients complained of headaches. Further, he noted insomnia in two, vomiting in two, diarrhœa in one, increased thirst in one, and sensation of heat in one case. In one of the cases of rheumatic blennorrhagia each dose of agathin was followed by increase of articular pains.

L. Badt's[2] experience corroborates in every respect the observations of Ilberg, at any rate as regards the evil after-effects. In the case of one female patient suffering from sciatica, for whom he had prescribed agathin in doses of half a gramme twice a day, headache and giddiness came on on the second day. The third day, having again taken half a gramme of agathin in the evening, she vomited and completely lost consciousness, an occurrence that had not previously happened. One is bound to hold the agathin responsible for these accidents. It is necessary, too, to state that the drug was without any curative effect in this case as in the others of chronic muscular rheumatism. This author is emphatic in his condemnation of agathin on account of the bad effects following its administration.

Laquer has reported a case of supra-orbital neuralgia in which salicylates in large doses had failed to give relief, which was relieved after three days' treatment with agathin. The patient passed a more restful night and the appetite improved. In four days the relief was complete, and no unpleasant after-effects were noticed. Rosenbaum had likewise an equally good result in a case of supra-orbital neuralgia.

[1] Deutsch. Med. Woch., No. 5, 1893, p. 119; les Nour. Rem. No. 10, 1893.

Deutsch Med. Woch., No. 15, 1893; les Nour. Rem., June 24th, 1893.

